# Induced Collagen Cross-Links Enhance Cartilage Integration

**DOI:** 10.1371/journal.pone.0060719

**Published:** 2013-04-04

**Authors:** Aristos A. Athens, Eleftherios A. Makris, Jerry C. Hu

**Affiliations:** 1 Department of Biomedical Engineering, University of California Davis, Davis, California, United States of America; 2 Davis Senior High School, Davis, California, United States of America; 3 Department of Orthopedic Surgery and Musculoskeletal Trauma, University of Thessaly (BIOMED), Larisa, Greece; University of Pittsburgh, United States of America

## Abstract

Articular cartilage does not integrate due primarily to a scarcity of cross-links and viable cells at the interface. The objective of this study was to test the hypothesis that lysyl-oxidase, a metalloenzyme that forms collagen cross-links, would be effective in improving integration between native-to-native, as well as tissue engineered-to-native cartilage surfaces. To examine these hypotheses, engineered cartilage constructs, synthesized via the self-assembling process, as well as native cartilage, were implanted into native cartilage rings and treated with lysyl-oxidase for varying amounts of time. For both groups, lysyl-oxidase application resulted in greater apparent stiffness across the cartilage interface 2–2.2 times greater than control. The construct-to-native lysyl-oxidase group also exhibited a statistically significant increase in the apparent strength, here defined as the highest observed peak stress during tensile testing. Histology indicated a narrowing gap at the cartilage interface in lysyl-oxidase treated groups, though this alone is not sufficient to indicate annealing. However, when the morphological and mechanical data are taken together, the longer the duration of lysyl-oxidase treatment, the more integrated the interface appeared. Though further data are needed to confirm the mechanism of action, the enhancement of integration may be due to lysyl-oxidase-induced pyridinoline cross-links. This study demonstrates that lysyl-oxidase is a potent agent for enhancing integration between both native-to-native and native-to-engineered cartilages. The fact that interfacial strength increased manifold suggests that cross-linking agents should play a significant role in solving the difficult problem of cartilage integration. Future studies must examine dose, dosing regimen, and cellular responses to lysyl-oxidase to optimize its application.

## Introduction

Because of articular cartilage's lack of inherent healing potential, lesions tend to degenerate to osteoarthritis (OA), a significant problem affecting over a third of adults aged 65 and over [1]. Currently, there are no cartilage treatments that offer long-term functionality. Mosaicplasty and microfracture require defect site preparation via cartilage removal. Subsequently, the defect is filled by either cartilage plugs or a “super clot” [2]. Autografts and allografts are also options. For these and other procedures, success is predicated upon the fill tissue's integration with native cartilage. Various strategies and materials have been proposed to integrate cartilage and bone [3]–[6]. However, cartilage-to-cartilage integration has proven to be notoriously difficult, even when using tissue engineering approaches [7], [8]. To achieve long-term, durable repair, grafts and engineered articular cartilage alike need to be integrated with native cartilage. Without proper integration, the implant will fall out of place or degrade rapidly [9], likely due to the high stress concentrations that occur at cartilage interfaces *in vivo*.

The general consensus regarding the main factors that hinder integration are: 1) Cell death, at the wound edge [8] and in surgically prepared defects, leads to metabolically inactive tissue, which prevents cell adhesion and migration to the injury site [10]–[14]. 2) Cell migration to the wound edge is hindered by the dense collagen network [10]–[14]; in native cartilage, cells are locked into lacunae and are not observed to migrate [15]. 3) Lack of cross-links between matrices of native and implant tissues [16], [17]. In short, the insufficiency of viable cells at the wound edge prevents synthesis of integrative matrix between the two surfaces to be joined [12]–[14], [18], [19], in part by lack of matrix synthesis. Even when viable cells are present, the newly synthesized matrix may not be sufficiently cross-linked to the native tissue. This study aims to overcome all of these factors by supplying viable cells to the interface via engineered neocartilage to mitigate the issues of cell death and lack of cell migration at the wound edge by exogenously inducing cross-links.

One way to deliver cells at an interface may be via the use of constructs engineered using the self-assembling process, which is an established method for generating tissue with abundant cells at the construct edge [20]. This method has also generated neocartilage with properties approaching those of native tissue [20]. Maintenance of cartilage with normal functional properties requires sustaining cell density; large areas of cell death would undoubtedly result in biomechanically inferior matrix or none at all [21]. Thus, this study seeks to use tissue engineered constructs created via chondrocyte self-assembly to deliver a higher cell density to the wound edge to enhance integration.

Another suggested mechanism for the enhancement of integration is collagen pyridinoline (PYR) cross-links [22]. PYR cross-links have been shown to be a major factor in determining the stiffness of connective tissues. PYR naturally forms within cartilage and other musculoskeletal tissues during development and aging via the enzyme lysyl oxidase (LOX), a metalloenzyme that converts amine side-chains of lysine and hydroxylysine into aldehydes. *In vivo*, LOX is most active at sites of growing collagen fibrils [1]. A potential method for inducing collagen cross-linking across cartilage interfaces is thus the exogenous application of this enzyme. Since LOX is a small-sized molecule, at roughly ∼50 kDa, and since cross-link formation occurs over several weeks, exogenous LOX can be applied to *in vitro* cultures on a continuous basis to ensure full penetration via diffusion and to allow sufficient time for cross-link formation. By employing LOX, one would expect the formation of "anchoring" sites, composed of PYR cross-links in the collagen network of the engineered tissue as well as of the native tissue, to bridge the two tissues together. Thus, LOX application combined with the delivery of high cell numbers to the wound edge are expected to promote tissue integration.

Using the self-assembling process, the objective of this study was to determine whether LOX can alter the integration of native-to-construct and native-to-native tissue systems through two experiments. It was hypothesized that application of LOX would enhance integration, as evidenced through tensile measurements. The first experiment sought to examine whether LOX would promote integration between native cartilage and neocartilage and to determine time and duration of application. The second experiment sought to determine whether the results from the first experiment can be replicated in a native-to-native cartilage system.

## Materials and Methods

### Cell and tissue harvest

Articular cartilage was harvested from distal femurs of one-week old male calves (Research 87 Inc., Boston, MA) less than 36 hr after sacrifice. To obtain the cells, following harvest, the tissue was digested in 0.2% collagenase type II (Worthington, Lakewood, NJ) in culture medium for 24 hr as previously described [23]. Culture medium formulation is as follows: DMEM with 4.5 mg/mL glucose and L-glutamine, 100 nM dexamethasone, 1% fungizone, 1% penicillin/streptomycin, 1% ITS+, 50 µg/mL ascorbate-2-phosphate, 40 µg/mL L-proline, and 100 µg/mL sodium pyruvate. Cell viability was assessed using trypan blue exclusion, and cells were frozen at −80°C using DMEM containing 20% fetal bovine serum (Atlanta Biologicals, Lawrenceville, GA) and 10% dimethyl sulfoxide until use. To reduce animal variability, cells from four animals were pooled together for cell seeding.

### Self-assembly of constructs

Cylindrical, non-adherent, agarose wells were prepared by placing 5 mm diameter stainless steel posts in 48 well plates filled with 1 ml of 2% molten agarose, as previously described [24]. After the agarose gelled, posts were removed. The resultant wells were saturated with two exchanges of medium. After thawing, cells were counted, viability was assessed using trypan blue exclusion, and cells were seeded into the agarose wells at a concentration of 5.5 million/100 µl medium. After 4 hr, an additional 400 µl of medium was added per well. To prevent disruption of the construct, complete medium change did not occur until after 24 hr. Constructs were cultured at 10% CO_2_, 37°C, in a humidified incubator for a total of t = 28 d (t = 1 d defined as 24 hr post seeding). Medium was changed daily (500 µl).

### Tissue integration

**Figure 1:**
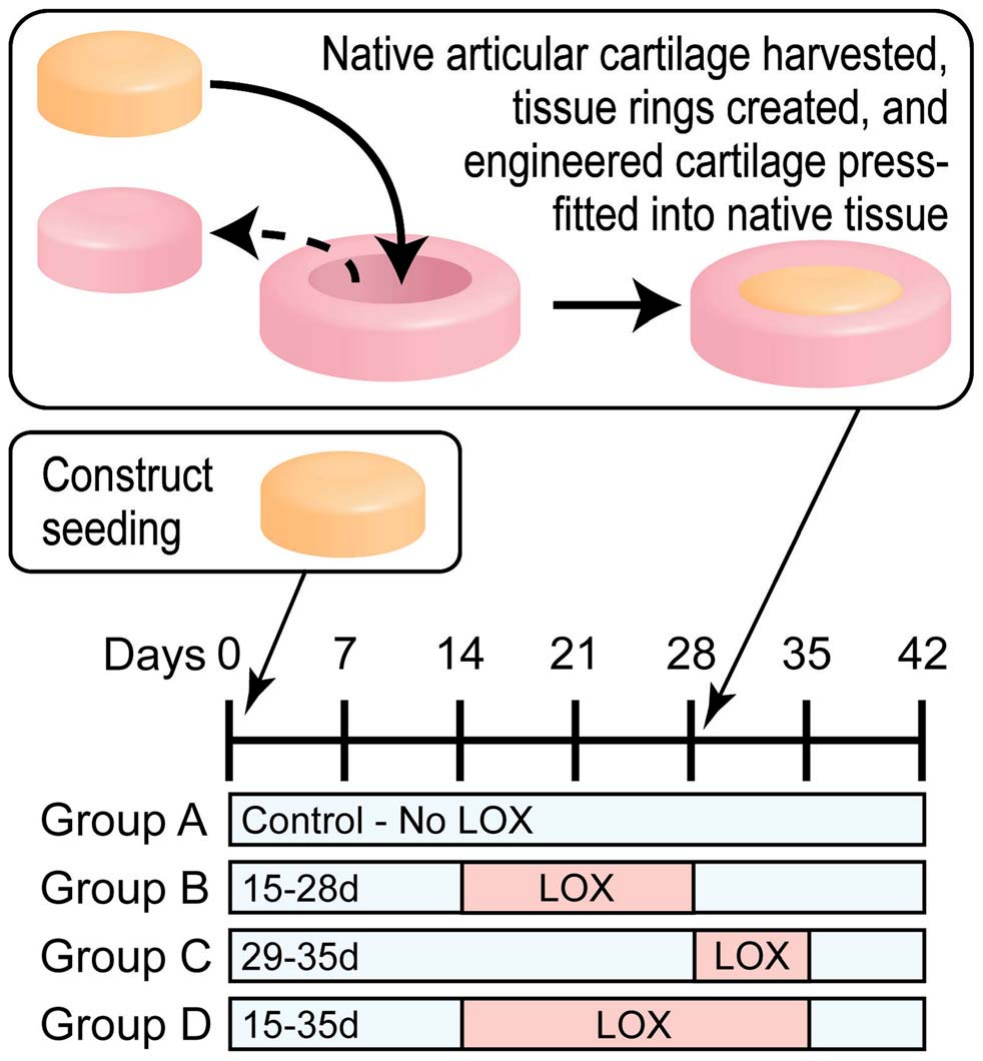
Schematic of the experiment examining integration of tissue engineered cartilage to native cartilage. For Group B, LOX was applied during construct formation, t = 15–28 d. For Group C, LOX was applied after forming the construct-to-native assemblies, t = 29–35 d. For Group D, LOX was applied both before and after the formation of the construct-to-native assemblies, t = 15–35 d.

To examine the study's hypotheses, two separate, but concurrent, experiments were conducted. First, the use of LOX was examined for the construct-to-native interface. At t = 28 d, engineered constructs were removed from culture and prepared for integration with native articular cartilage. The t = 28 d culture time was chosen to coincide with prior work in self-assembled cartilage and with other cartilage tissue engineering efforts. Bovine articular cartilage explants, measuring 6 mm×1 mm, were harvested using biopsy punches. A concentric, 4 mm diameter defect was punched from the explant. From the engineered constructs, 4 mm diameter biopsies were obtained and press-fitted into the defect in the explant (Fig. 1). To ensure that all constructs were in firm contact with the explants, cyanoacrylate was applied; a penetration depth of 25 µm (∼2.5% of thickness) and degradation within the culture period were verified using histology. These construct/explant assemblies were cultured for an additional 14 d, at which point they were removed for assessments. The second experiment consisted entirely of explants instead of constructs. Native-to-native tissue assemblies were formed using the same methods as described above.

### Collagen cross-linking via lysyl oxidase

The LOX medium contained a concentration of 0.15 µg/ml LOX (GenWay Biotech, Inc., San Diego, CA). This concentration is based a pilot study in which three concentrations, 0.0015, 0.015, and 0.15 µg/ml of LOX, were examined. The results showed that only 0.15 µg/ml of LOX improved pyridinoline content over the culture duration employed; at this concentration, neither the collagen nor glycosaminoglycan per wet weight (collagen/ww and GAG/ww, respectively) was altered when LOX was applied to either native or engineered cartilage separately. For the construct-to-native study, four groups were examined: The control (Group A) consisted of construct/explant assemblies maintained in culture medium only. Group B was treated with the LOX medium during t = 15–28 d. Group C was treated during t = 29–35 d. Group D was treated during t = 15–35 d. These groups were chosen to examine LOX treatment prior (Group B), after (Group C), or throughout (Group D) integration with the native tissue. Assemblies were assessed at t = 42 d to allow for a total of 14 d of integration time. For the native-to-native study, two groups were examined. The control group was allowed to integrate for 14 d in culture medium, while the LOX Group was maintained in a LOX medium during the same time.

### Histology

Frozen sections were collected at 14 µm on positively charged slides to promote maximal adherence. These were fixed in 10% neutral buffered formalin and stained. Picrosirius red was used to demonstrate collagen distribution, as previously described [20]. After staining, slides were dehydrated through ascending alcohol percentages (50%, 60%, 70%, 80%, 90%, 95%, 100%) to minimize dehydration artifacts, and coverslips were applied using Permount.

### Biochemistry

Total collagen was assessed using a hydroxyproline assay, and glycosaminoglycan (GAG) content was measured using a Blyscan kit, both as previously described [20].

### Tensile testing

Assemblies were cut into strips 1 mm wide. Thickness and width were verified photographically using ImageJ (National Institutes of Health, Bethesda, MD). Specimens were glued onto test strips separated by a pre-defined spacing of 1 mm, and the strips were clamped and exposed to constant uniaxial strain of 1% of the 1 mm spacing per second until failure using a uniaxial materials testing machine (Instron 5565). Force-deformation data were collected and then normalized with respect to the cross-sectional area and initial spacing length of the specimens. From this, an apparent “stiffness” was derived by calculating the slope of the linear region of the graph. The ultimate tensile apparent strength (UTS) was defined as the maximum stress attained by the specimen before failure.

### Statistical analysis

Based on prior data used to determine LOX concentration, application time, and effects on cellular activity, a power analysis was performed to determine an n = 6 required to discern differences in tensile properties at p<0.05. Data were compiled as mean±standard deviation and analyzed using a single factor ANOVA. If the F-test was statistically significant, a Tukey's *post hoc* test was employed to identify significant groups. Significance was defined as p<0.05.

## Results

### Integration of engineered constructs to native articular cartilage

**Figure 2:**
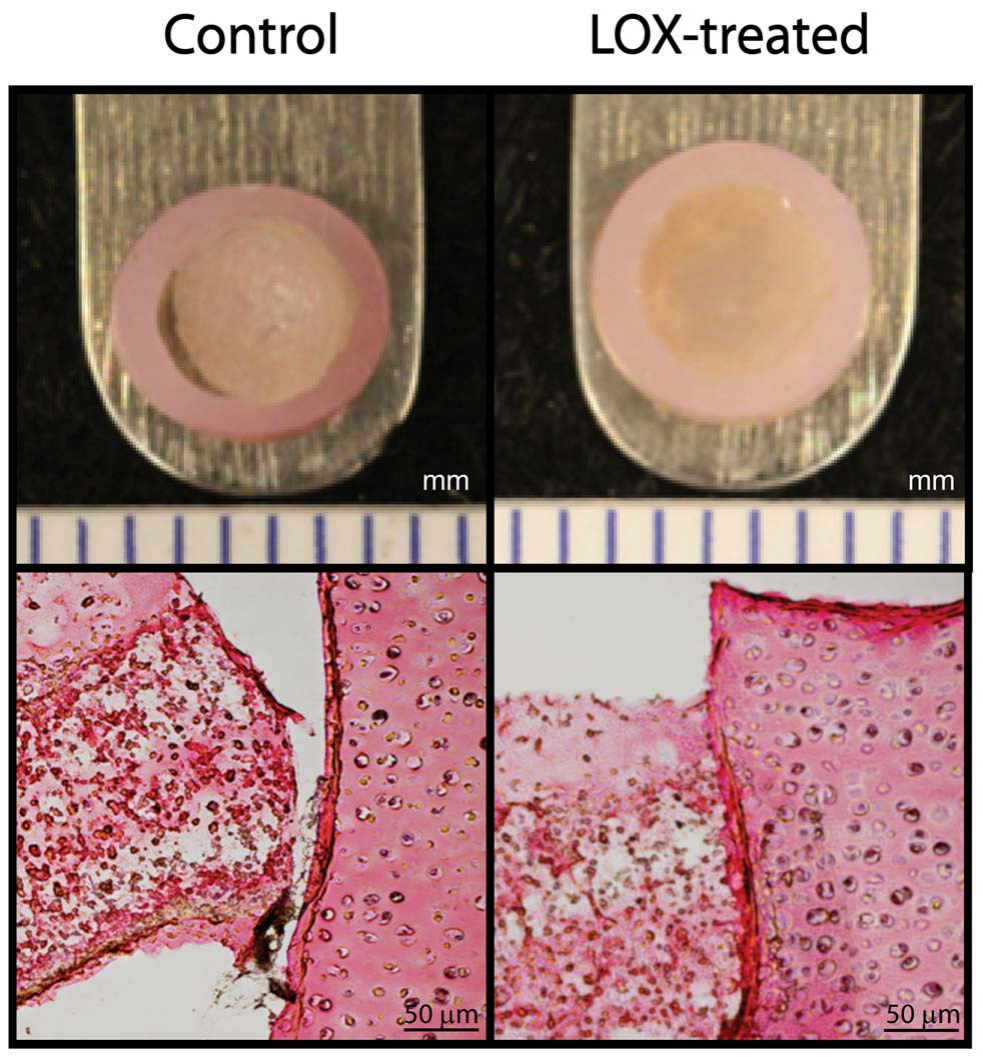
Gross morphology and histology of constructs/explant assemblies. Straight from culture, most controls resembled LOX-treated samples, though gaps were seen in one-third of the controls (upper left panel). None of the LOX-treated samples displayed gaps that were grossly visible; a representative sample (Group D) is shown (upper right). Gaps in the controls were also seen after histological processing using picrosirius red (lower left) versus LOX-treated samples (lower right, Group D).

**Figure 3:**
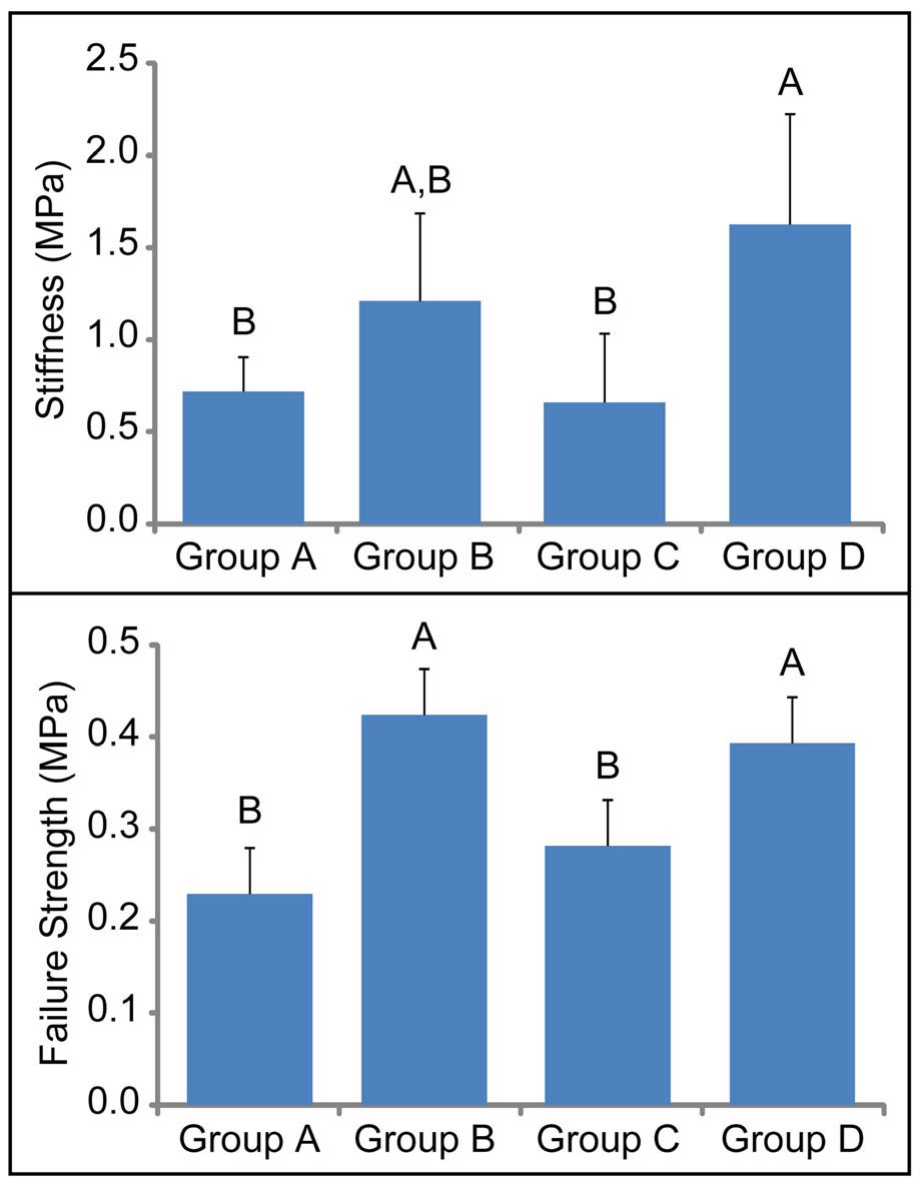
Tensile mechanical data of construct/explant interface. Significantly higher apparent stiffness (top) was seen when LOX was applied during t = 15–35 d (Group D) than controls (Group A). Significantly higher apparent strength was obtained across the integration interface when engineered cartilage was treated with LOX before being press-fitted into the native cartilage (bottom). Bars with different letters are significantly different (p<0.05).

For all treatment durations, LOX-treated construct-to-native assemblies displayed better integration as compared to controls using gross morphology, histology, and biomechanical evaluations. Prior to histological processing, the assemblies were evaluated straight from culture for gross morphology. Although LOX addition increased the stiffness of the assemblies, it did not affect the size and dimensions of the samples. Grossly, gaps were seen between the construct and native tissue in 33% of the controls (Fig. 2). Gaps were not seen for any of the LOX-treated specimens. Similarly, histological evaluation showed gaps in the controls, while LOX-treated samples showed construct adherence to the native tissue. Tensile testing across the integration interface showed significantly higher apparent stiffness when LOX was applied during t = 15–35 d (Group D) (1.6±0.6 MPa, versus control values of 0.7±0.2 MPa (Fig. 3, top)). Significantly higher apparent strength values were observed for both Groups B and D (0.42±0.07 MPa and 0.39±0.06 MPa, respectively), where LOX was applied before formation of the construct-to-native assembly (Fig. 3, bottom). Control and Group C values were 0.23±0.08 MPa and 0.28±0.1 MPa. No significant differences were detected in the GAG/ww or collagen/ww among the construct or the explant portions of the assemblies. Specifically, no significant differences were detected in the GAG/ww content among the constructs (4.5±1.4%, 2.9±1.4%, 3.1±0.5%, and 4.5±0.6% for Groups A–D, respectively) or among the explant rings (8.6±1.1%, 10.9±1.9%, 9.6±01.2%, and 10.5±2.8% for Groups A-D, respectively). Additionally, no significant differences were detected in the collagen/ww content among the constructs (5.4±2.2%, 5.7±1.7%, 5.7±0.7%, and 7.2±3.1% for Groups A-D, respectively) or among the explant rings (5.6±3%, 10.6±7.5%, 6.2±3%, and 10.5±3.2% for Groups A-D, respectively).

### Integration between native cartilage tissues

**Figure 4:**
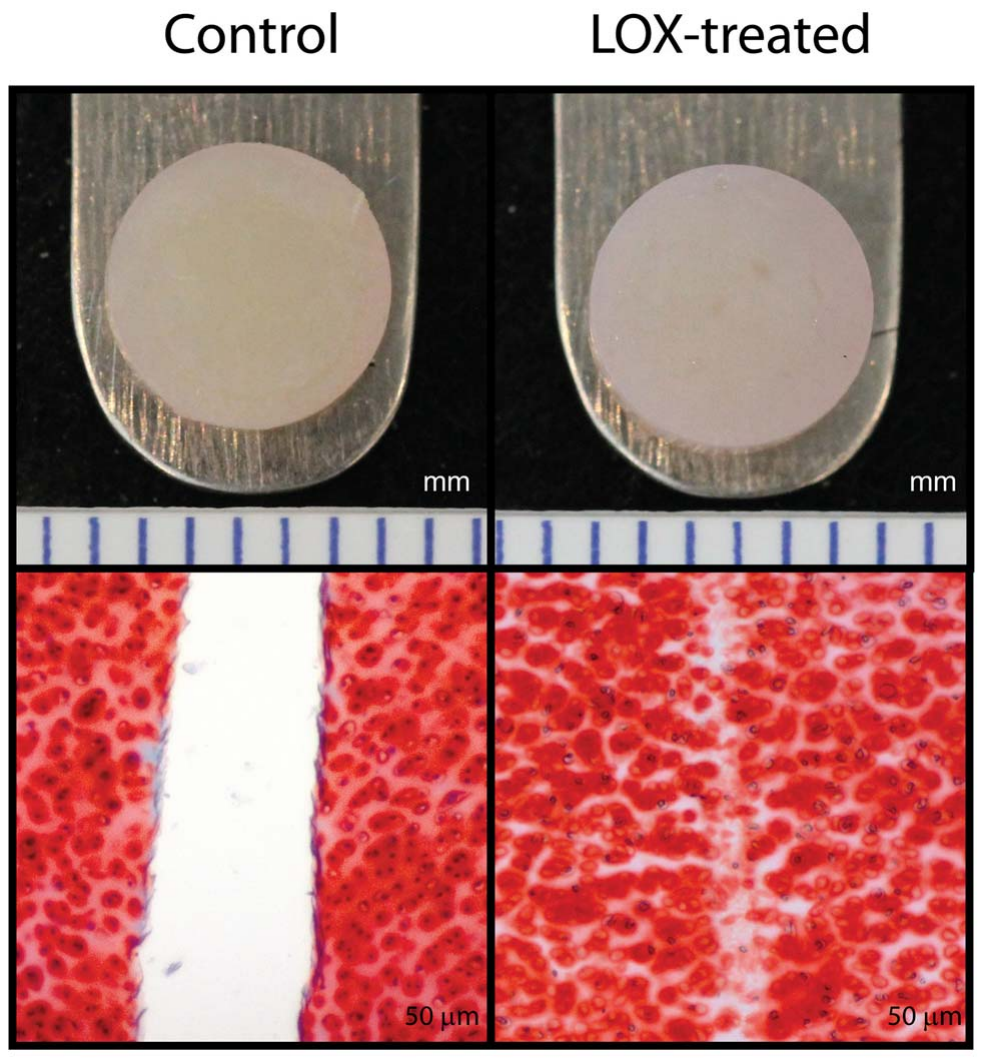
Gross morphology and histology of explant/explant assemblies. Neither control nor LOX-treated native-to-native assemblies displayed grossly visible gaps when removed from culture (top row). However, gaps can be seen after histological processing using picrosirius red in the control group, unlike the LOX-treated group (bottom).

**Figure 5:**
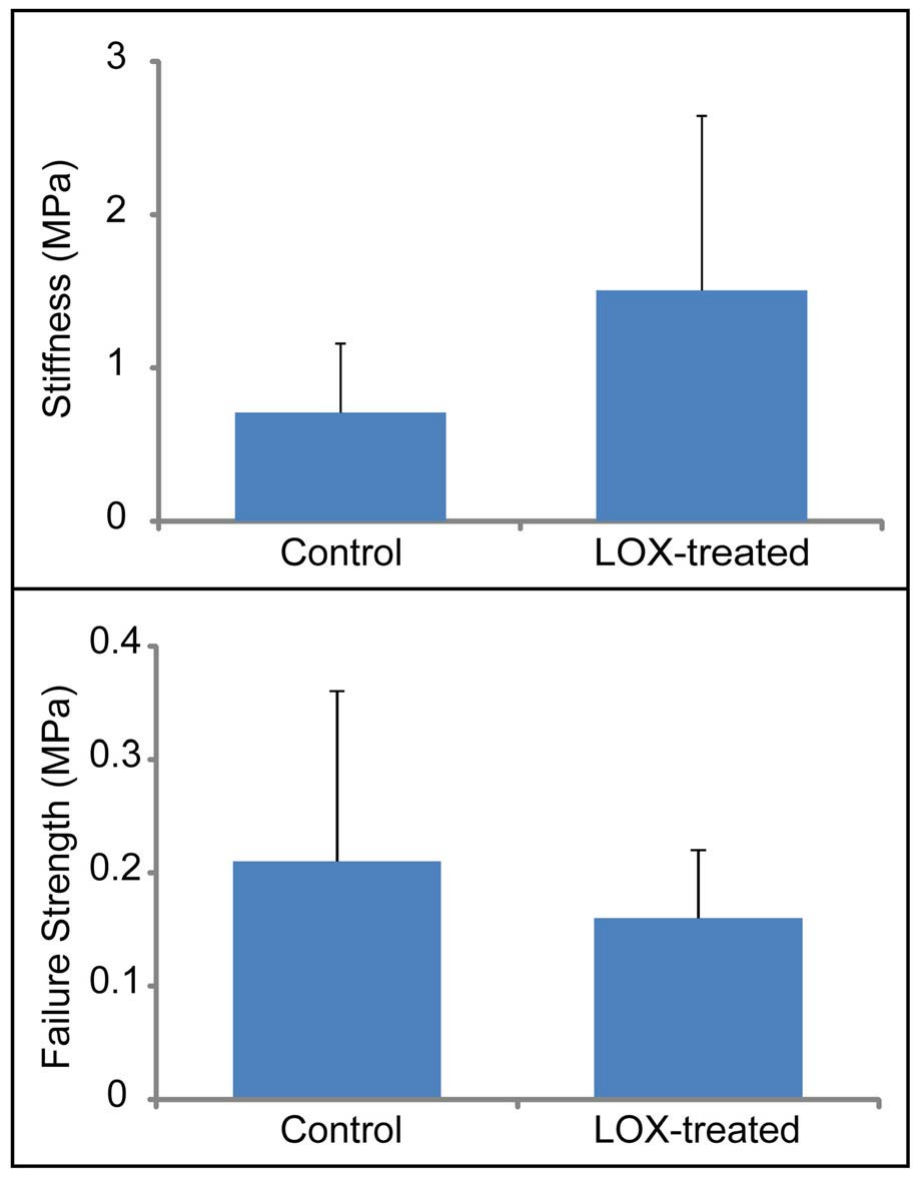
Tensile mechanical data of explant/explant interface. No differences in apparent stiffness (top) or apparent strength (bottom) were seen when LOX was applied to native-to-native integration.

Qualitatively, the only difference between control and LOX-treated native-to-native assemblies was seen by histology (Fig. 4). The apparent stiffness of the LOX-treated group was more than twice that of the control (1.5±1.1 MPa versus 0.7±0.4 MPa), though neither this property nor the apparent strength were statistically significant (Fig. 5). This is potentially due to biological variations, since, in contrast with the engineered tissues which were formed using cells pooled from multiple animals, each native-to-native assembly is derived from a different animal. GAG and collagen content were not different between the two groups.

## Discussion

Motivated by the as-of-yet unsolved issue of cartilage integration, the objective of this study was to examine the hypothesis that LOX would induce cartilage integration. This enzyme naturally occurs in cartilage and promotes PYR cross-links in collagen, thereby holding potential for strengthening cartilage-to-cartilage interfaces. The hypothesis was proven to be correct as evidenced by the biomechanical and histological data. At the dosage applied, this naturally occurring enzyme did not alter cellular response with respect to collagen and GAG production. Engineered tissues, formed using a self-assembling process, were integrated to native tissue explants by applying LOX to a ring-and-implant assembly (Fig. 1). Additionally, LOX was applied to native-to-native cartilage interfaces to examine whether this novel integration method can also be applicable to cases where there is not an abundance of cells at the wound edge. The results showed that cartilage integration can be enhanced if the interface is stocked with metabolically active cells and PYR cross-links simultaneously. Enhanced interfacial properties were observed for construct-to-native but not for the native-to-native case. Group D, which was treated with LOX for the longest period of time, had statistically higher tensile properties at the interface than did the other three groups. Specifically, Group D had approximately 2.2 times the tensile strength of controls. This was confirmed with morphological and histological data. The results of this study are significant for both current and prospective cartilage regeneration and repair methods.

It is worth noting that, despite the lack of any significant differences in the collagen and GAG content in either the construct or explant groups, there were significant LOX-induced increases in interface biomechanics. The fact that interfacial mechanical properties (apparent stiffness and apparent strength) increased significantly in the absence of increases in the main extracellular matrix (ECM) components suggests that cross-links play a central role in integration. Unfortunately, a relationship between the strength of the interface and the number of cross-links at the interface cannot be directly assessed. This is because the interface cannot be isolated without adjacent tissues that, too, contain cross-links. It is, therefore, difficult to ascertain the fraction of cross-links belonging to the interface alone. The same can be said of the collagen and GAG production by chondrocytes at the interface. Since the interface consists of a thin layer, minute changes in the ECM of this area would be masked by the comparatively greater ECM content of the cartilages undergoing integration. The mechanism of LOX-induced collagen cross-linking is well-established [22] and a strong candidate for explaining the results obtained in this study, though this was not directly proven here. Bolstering this hypothesis, recent studies have shown induced collagen crosslinks in engineered cartilage improves tensile stiffness [25], [26]. Future studies may consider techniques such as time-resolved fluorescence spectroscopy [27] to quantify PYR at the interface.

It was observed during tensile testing that samples always broke at the interface, indicating that the interface is not as strong as either neocartilage or native cartilage. The mechanical property of the interface is, thus, due to newly synthesized matrix that has had relatively little time to develop cross-links in contrast to the rest of the tissues. It is known that LOX-induced PYR formation requires 7 to 30 days [28], which may also explain why increased apparent strength was observed only for groups whose LOX treatment was initiated 28 d prior to tensile assessment. In these cases, PYR cross-link precursors were allowed to accumulate within the constructs prior to their being press-fitted into the native tissue, at which time these precursors readily bridged the construct and native tissue together by maturing into cross-links. In terms of native-to-native interfaces, it may be prudent to consider longer durations of LOX application in future studies.

It is worthy to note that, for the timescale applied, diffusion of LOX should not be a bottleneck to its effectiveness. LOX is a relatively small molecule of ∼50 kDa. For comparison, BMP-1 is 30 kDa, and the diffusion coefficient of 40 kDa dextran has been determined to be ∼60 µm^2^/s) [29]. However, LOX may require time to act before it promotes integration since it can take weeks to complete the final PYR product. This can be seen with Group C, which consists of LOX applied at t = 25–39 d only. This treatment did not result in significant increases in tensile properties. It is unclear whether this is due 1) to the short duration of LOX application or 2) to the late initiation of application. These two variables should be examined in a future study at greater detail. For example, a variety of initiation and culture times can be examined, extending the total time of culture up to 8 weeks to identify the "ceiling" of effectiveness. Once this saturation level has been determined, one can then optimize not only the time of initial application, but also the total duration of application.

Aside from the dependence on *in vitro* culture time [30], cytokines present *in vivo* can also influence integration. A study examined the effects of steroid hormones in bovine cartilage that is lacking a known inhibitor to integration, interleukin-1β. An increase of ∼50 kPa in mechanical integration was seen [31], as compared to the 700 kPa obtained in this study for the native-to-native controls. Also, it has also been shown that, without the assistance of exogenous agents, strength of half that which is seen in intact cartilage can be achieved in an equine model for chondrocyte transplantation [32]. It is worth noting that, in the present study, by delivering cells to the interface in concert with LOX, integration strength can be increased to 1.6 MPa (Group D). Comparing this result to the stiffness of fibrin, which is clinically used as tissue glue and sealant, the stiffness of the LOX-treated interface is roughly fifty times higher. Averaged over various strain rates, the stiffness of fibrin alone is under 30 kPa.[33] When fibrin is combined with chondrocytes to serve as a cartilage adhesive, the stiffness of the interface is increased over fibrin alone and also with time *in vivo*, to 0.645 MPa after 8 months [11]. It is worth noting that LOX-treatment achieves two-times the stiffness in a fraction of the time. It is expected that the stiffness of interfaces enhanced with LOX and chondrocytes will continue to improve *in vivo* as the cells remodel the matrix over time. Chondrocyte transplantation is a current therapy that, similar to the self-assembled constructs employed in this study, delivers metabolic cells to the wound edge using fibrin. It is conceivable for LOX to assist this clinical procedure, especially since the LOX treatment produces comparable results to fibrin at a shorter time. Of course, additional studies on 1) optimal dosing time, 2) cross-linker concentration, and 3) activity profile as related to not only the chondrocytes but also other cell types surrounding cartilage would need to be completed to ensure safety and efficacy, prior to deploying this technique clinically.

A major component of articular cartilage ECM is the electronegative aggrecan. This electric charge is an obstacle to integration because the similar charges in two pieces of tissue would cause them to repel [7]. Further studies need to be completed to fully understand the role which aggrecan's electronegativity may play in blocking integration. Future studies might also include the combination of LOX with other bioactive agents that are known to influence cartilage behavior. Already, transforming growth factor β1 (TFG-β1) has shown efficacy when combined with a biomaterial [34], and it would be interesting to examine how LOX can assist this case. TFG-β1 may work in synergism with LOX, the cytokine and enzyme working in tandem to effect greater collagen production and cross-linking.

It should be mentioned that, for this study, LOX concentration was based on a pilot study that examined LOX on native and engineered cartilages separately (described in “Materials and Methods”). Following this study's exciting results, it may be prudent to conduct a systematic examination of various LOX concentrations to identify a minimum, yet effective, concentration between 0.015 and 0.15 µg/ml that enhances interfacial stiffness and strength to the levels of the engineered or native cartilages, or even for other tissues where cross-linking plays important functional roles. For instance, integrating engineered knee meniscus to native knee meniscus has shown dependence on maturation state [35], and therefore the extent of collagen cross-links, and LOX may be used similarly for this tissue. Finding this minimum dose will be significant in not only reducing cost but also in mitigating any potential for this enzyme to interfere with other cellular processes, despite this being a naturally-occurring enzyme. Already, it has been shown here that LOX does not interfere with chondrocyte metabolism with respect to collagen and GAG synthesis, but, for its use *in vivo*, the effects of LOX on other cells may need to be elucidated prior to conducting animal studies with this enzyme. A similar process would allow the identification of an optimal LOX concentration for maximizing the native-to-native integration strength; this will be immensely useful from a clinical perspective, once the safety and efficacy of exogenous LOX has been shown.

While other cross-linkers such as ribose, glutaraldehyde, genipin, and methylglyoxal have all been investigated in conjunction with engineered articular cartilage [36], [37], these agents have all been shown to alter cellular activity. Some of these agents are even cytotoxic and thus preclude their use with live cells in influencing integration. Furthermore, unnatural cross-linkers such as glutaraldehyde have been shown to elicit a foreign body giant cell reaction [38], in contrast to LOX, which is found naturally in multiple musculoskeletal tissues. This study demonstrates that LOX is a potent agent for enhancing integration between native and tissue engineered cartilage. It also paves the way for the use of LOX in improving native cartilage integration. These results could potentially be used to solve the problem of large cartilage defects by allowing tissue engineered cartilage implants to be integrated into the surrounding tissue.
